# TurboID mapping reveals the exportome of secreted intrinsically disordered proteins in the transforming parasite *Theileria annulata*

**DOI:** 10.1128/mbio.03412-23

**Published:** 2024-05-15

**Authors:** Francis Brühlmann, Carmen Perry, Charlotte Griessen, Kapila Gunasekera, Jean-Louis Reymond, Arunasalam Naguleswaran, Sven Rottenberg, Kerry Woods, Philipp Olias

**Affiliations:** 1 Institute of Animal Pathology, University of Bern, Bern, Switzerland; 2 Department of Chemistry, Biochemistry and Pharmaceutical Sciences, Bern, Switzerland; 3 Institute of Veterinary Pathology, Justus Liebig University, Giessen, Germany; University of Geneva, Geneva, Switzerland

**Keywords:** protozoa, *Toxoplasma*, *Plasmodium*, *Cryptosporidium*, cancer, theileriosis, malaria, *Babesia*, East Coast fever, neglected tropical disease, cattle, BioID

## Abstract

*Theileria annulata* is a tick-transmitted apicomplexan parasite that gained the unique ability among parasitic eukaryotes to transform its host cell, inducing a fatal cancer-like disease in cattle. Understanding the mechanistic interplay between the host cell and malignant *Theileria* species that drives this transformation requires the identification of responsible parasite effector proteins. In this study, we used TurboID-based proximity labeling, which unbiasedly identified secreted parasite proteins within host cell compartments. By fusing TurboID to nuclear export or localization signals, we biotinylated proteins in the vicinity of the ligase enzyme in the nucleus or cytoplasm of infected macrophages, followed by mass spectrometry analysis. Our approach revealed with high confidence nine nuclear and four cytosolic candidate parasite proteins within the host cell compartments, eight of which had no orthologs in non-transforming *T. orientalis*. Strikingly, all eight of these proteins are predicted to be highly intrinsically disordered proteins. We discovered a novel tandem arrayed protein family, nuclear intrinsically disordered proteins (NIDP) 1–4, featuring diverse functions predicted by conserved protein domains. Particularly, NIDP2 exhibited a biphasic host cell-cycle-dependent localization, interacting with the EB1/CD2AP/CLASP1 parasite membrane complex at the schizont surface and the tumor suppressor stromal antigen 2 (STAG2), a cohesion complex subunit, in the host nucleus. In addition to STAG2, numerous NIDP2-associated host nuclear proteins implicated in various cancers were identified, shedding light on the potential role of the *T. annulata* exported protein family NIDP in host cell transformation and cancer-related pathways.

**IMPORTANCE:**

TurboID proximity labeling was used to identify secreted proteins of *Theileria annulata*, an apicomplexan parasite responsible for a fatal, proliferative disorder in cattle that represents a significant socio-economic burden in North Africa, central Asia, and India. Our investigation has provided important insights into the unique host-parasite interaction, revealing secreted parasite proteins characterized by intrinsically disordered protein structures. Remarkably, these proteins are conspicuously absent in non-transforming *Theileria* species, strongly suggesting their central role in the transformative processes within host cells. Our study identified a novel tandem arrayed protein family, with nuclear intrinsically disordered protein 2 emerging as a central player interacting with established tumor genes. Significantly, this work represents the first unbiased screening for exported proteins in *Theileria* and contributes essential insights into the molecular intricacies behind the malignant transformation of immune cells.

## INTRODUCTION

The apicomplexan phylum harbors diverse parasites, among which *Plasmodium*, *Toxoplasma*, *Cryptosporidium*, and *Theileria* stand out for their impact on human and animal health. Apicomplexans use sophisticated mechanisms to manipulate host cells, inducing metabolic shifts and changes in host gene expression (1). Transforming *Theileria* species orchestrate profound host cell changes that resemble cancerous cell phenotypes (2–4). The resulting disease in cattle, prevalent in the Southern Hemisphere and parts of Asia, is characterized by fever, anemia, and massive lymph node enlargements mirroring lymphoma, with a high mortality rate and a devastating impact on local farming communities (5–8). *Theileria*-induced transformation, which shares many similarities with cancer including uncontrolled proliferation, invasiveness, and metastasis, depends on the presence of a viable parasite (9), making the intricate parasitic manipulation of basic cell biological signaling pathways in the host an intriguing system to study. In this study, we focused on *T. annulata*, the causative agent of tropical theileriosis in cattle, which infects macrophages and B cells that undergo extensive post-infection modifications (10).

Little is currently understood about the mechanisms of how *T. annulata* manipulates host signaling pathways post-invasion, contributing to uncontrolled proliferation. In *Plasmodium* and *Toxoplasma*, the release of effector proteins before, during, and after invasion is critical to host cell entry and manipulation (11, 12). By contrast, the release of microsphere and rhoptry proteins by *Theileria* appears to occur only after the complete internalization of infectious sporozoites (13). Interestingly, and unlike other apicomplexan zoites, the *Theileria* sporozoite does not need to reorient itself to bring its apical pole into close contact with the host cell membrane but enters the host cell in any direction through a progressive so-called “circumferential zippering mechanism” (14). After the invasion process, *Theileria* parasites only briefly remain inside a parasitophorous vacuole membrane (PVM), from which they escape within minutes of infection to lie free in the host cell cytoplasm (15). Immediately after exiting the PVM, the parasite associates with host cell microtubules (13, 16). The unique positioning of *Theileria* parasites inside the host cell cytoplasm, not enclosed by a PVM, facilitates the recruitment, manipulation, and hijacking of host cell proteins such as end-binding protein 1 (EB1), CLIP-170-associating protein 1 (CLASP1), and IκB kinase (IKK) directly on their membrane surface (4, 16–18), distinguishing them from the other apicomplexan parasites. Approximately 3 to 4 days after leukocyte invasion by tick-borne sporozoites, the multinucleated schizont triggers uncontrolled clonal proliferation and host cell immortalization (19, 20), with the unique feature that each subsequent host cell division results in an equal distribution of the *Theileria* schizont between the two daughter cells (16, 21, 22). The transformed parasitized host leukocytes eventually start to spread throughout the lymphoid system and the rest of the body (23, 24). Within the parasite’s life cycle, the schizont stage plays a central role in the pathology associated with malignant *T. annulata*, setting it apart from other *Theileria* species such as non-malignant *T. orientalis* strains. In *T. orientalis,* the intra-erythrocytic piroplasm stage is responsible for the pathology, and no host cell transformation occurs (25, 26).

Infected cells in malignant theileriosis exhibit significant changes in gene expression profiles, including the persistent activation of the phosphatidylinositol 3-kinase (PI3-K) pathway (27), upregulation of c-Jun NH2-terminal kinase (JNK) (28, 29), increased c-Myc expression (30), and suppression of p53 activity (31, 32). The schizont hijacks the IKK signalosome on its membrane, activating the NF-κB pathway and influencing anti-apoptotic gene expression (17). Host manipulation induced by *T. annulata* is likely to involve the secretion of various effector proteins, which affect host signaling pathways and contribute to uncontrolled proliferation, invasiveness, and resistance to apoptosis (33). Despite extensive studies on parasite-induced changes to the host cell, only a limited number of exported proteins, including TaPIN1 (34), TaPHB (35), TashAT2 (36), TashHN (37), and Ta9 (38) have been identified. These effector proteins are suggested to be associated with specific host signaling changes that potentially contribute to uncontrolled and invasive cancer-like host cell behavior (38–42). However, a comprehensive understanding of the essential exportome responsible for the malignant alteration of the host cell is still lacking.

We employed an unbiased approach, using TurboID-based proximity labeling (43) of *T. annulata*-infected cells (TaC12), aiming to identify secreted effector proteins in the host cell cytoplasm and nucleus. This strategy revealed a novel gene family named nuclear intrinsically disordered proteins (NIDP1–4), alongside members of the Tash and Ta9 protein families. Antibodies against NIDP1–4 confirmed their nuclear localization. Remarkably, this gene family is absent in the non-transforming parasite *T. orientalis*. Detailed analysis of NIDP2 showed a biphasic localization pattern of the protein, accumulating in the host cell nucleus during interphase and associating with a protein complex at the schizont surface during mitosis. Within the nucleus, NIDP2 localized to the host chromatin and interacted with the tumor suppressor STAG2, shedding light on potential mechanisms underlying host cell transformation of cancer-related pathways. Our study provides a novel perspective on the shared characteristics of secreted *T. annulata* proteins, most strikingly their predicted intrinsic disorderedness, offering insights into their role in host cell manipulation.

## RESULTS

### TurboID-based proximity ligation in the host cell leads to the identification of secreted *Theileria annulata* proteins

**Fig 1:**
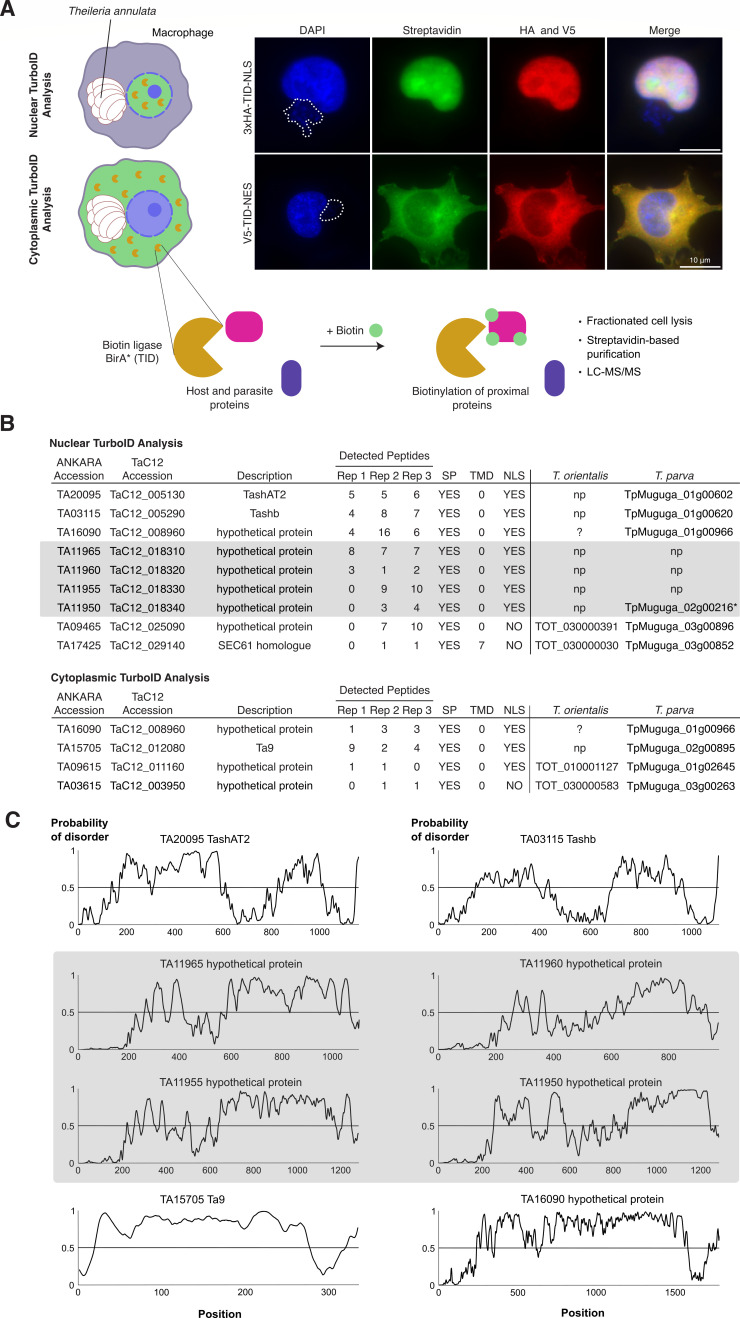
TurboID identifies proteins secreted by *T. annulata* into the host cell. (**A**) Schematic representation of TurboID (TID) approach in *Theileria annulata* TaC12 cells (infected macrophages). The biotin ligase TurboID was targeted to the host cell nucleus and cytoplasm. Upon the addition of biotin for 3 h, biotinylated host and parasite proteins were fractionated and analyzed by mass spectrometry. To verify the correct localization and activity, HA-TID-NLS-construct transduced (host nuclear TID) and V5-TID-NES construct transduced (host cytoplasmic TID) TaC12 cells were grown in the presence of biotin, fixed and analyzed by immunofluorescence assay using anti-HA or anti-V5 antibodies with additional staining of biotinylated proteins by FITC-conjugated streptavidin. The host cell nuclei and parasite schizont nuclei (indicated by a dotted line) are labeled with DAPI. See also Fig. S1. (**B**) Mass spectrometry results of three biological replicates from nuclear and cytoplasmic TID experiments with peptide counts of identified *T. annulata* proteins. Shown are proteins identified at least twice with a predicted signal peptide (SP) or transmembrane domain (TMD). See also Table S1 for the entire list. Highlighted in gray is a newly identified protein family with absent orthologs in non-transformative *T. orientialis*. Putative orthologs lacking a predicted SP in *T. orientalis* and *T. parva* are indicated with a star symbol. (**C**) Protein disorder score (IUPred3) of all identified proteins with no orthologs in *T. orientalis*. NLS, nuclear localization sequence; np, not present.

We performed TurboID-based proximity labeling in the host cell cytoplasm and nucleus, to identify proteins secreted by the parasite that might be involved in host cell transformation. For this, the promiscuous biotin ligase TurboID (43) was fused either to a nuclear localization signal (NLS) or a nuclear export signal (NES) sequence and expressed in the *T. annulata*-infected TaC12 macrophage cell line (Fig. 1A). By adding biotin to the cell culture media, proteins near the biotin ligase enzyme were biotinylated, affinity purified with streptavidin, and subsequently identified by mass spectrometry (LC-MS/MS). Immunofluorescence analysis (IFA) confirmed the correct localization of both fusion proteins in the continuously parasitized cell line TaC12 (Fig. 1A; Fig. S1A). Non-biotin controls were used for comparison and analyzed in parallel with three biological replicates for each construct. As an initial step in identifying new effector candidates, the detected peptide count in LC-MS/MS of the control was compared with the biotinylated samples. Based on these results, candidate proteins were prioritized according to the following criteria: The protein must occur in at least two of three replicates and contain a predicted signal peptide (SP) according to predictions of the SignalP 4.1 algorithm used with SignalP 3.0 sensitivity (44). These stringent criteria lead to nine candidate proteins predicted to be exported to the host nucleus, and four candidate proteins in the host cytosol (Fig. 1B). The full set of 179 *T*. *annulata* proteins identified with at least one peptide in at least one replicate in the nucleus (*n* = 105) and cytoplasm (*n* = 74), respectively, is provided in Table S1. We then searched for orthologs in the non-transformative species *T. orientalis* (45), hypothesizing the absence of orthologous proteins in case of importance for transformation. A BLAST analysis of the nine proteins identified in the nuclear fraction found that two proteins, TA09465 and TA17425, share sequence identities with *T. orientalis* proteins TOT_030000391 and TOT_030000030, respectively. In the cytosolic fraction, TA09615 and TA03615 were found to share identities with TOT_010001127 and TOT_030000583, respectively. We previously showed that TA03615 is associated with the parasite membrane (16). TA16090 was identified in both nuclear and cytosolic fractions, and while no clear ortholog in *T. orientalis* is detected, some similarity (29% identity) is found in the N-terminal part of the protein with the *T. orientalis* protein TOT_010000916.

Among the proteins in the host cell nuclear fraction with no orthologs in *T. orientalis*, we identified TashAT2 (TA20095) and Tashb (TA03115) (Fig. 1B). TashAT2 and Tashb are both members of the large Tash gene family clustered in tandem repeats on chromosome 1 (46) (Fig. S1D). We raised an antibody against the so far uncharacterized Tashb protein and confirmed that Tashb is targeted to the host cell nucleus of schizont-infected cells (Fig. S1B). TashAT2 has been previously identified as a secreted protein located inside the host cell nucleus of *T. annulata* D7 and TBL20 cell lines (36). We confirmed the nuclear localization of TashAT2 in TaC12 cells by IFA (Fig. S1C). Ta9 (TA15705), a member of the Ta9 gene family, is detected in the host cytoplasm with no orthologs in *T. orientalis* (Fig. 1B) (47, 48). We confirmed the anticipated localization in the host cytoplasm in TaC12 cells by IFA (Fig. S1E). Ta9 has previously been shown to be secreted into the host cell in the *T. annulata*-infected cell line Pendik and is suggested to be involved in AP-1 transcription factor (TF) activation when overexpressed in embryonic kidney cells (38).

### Identification of a novel protein family of secreted proteins

**Fig 2:**
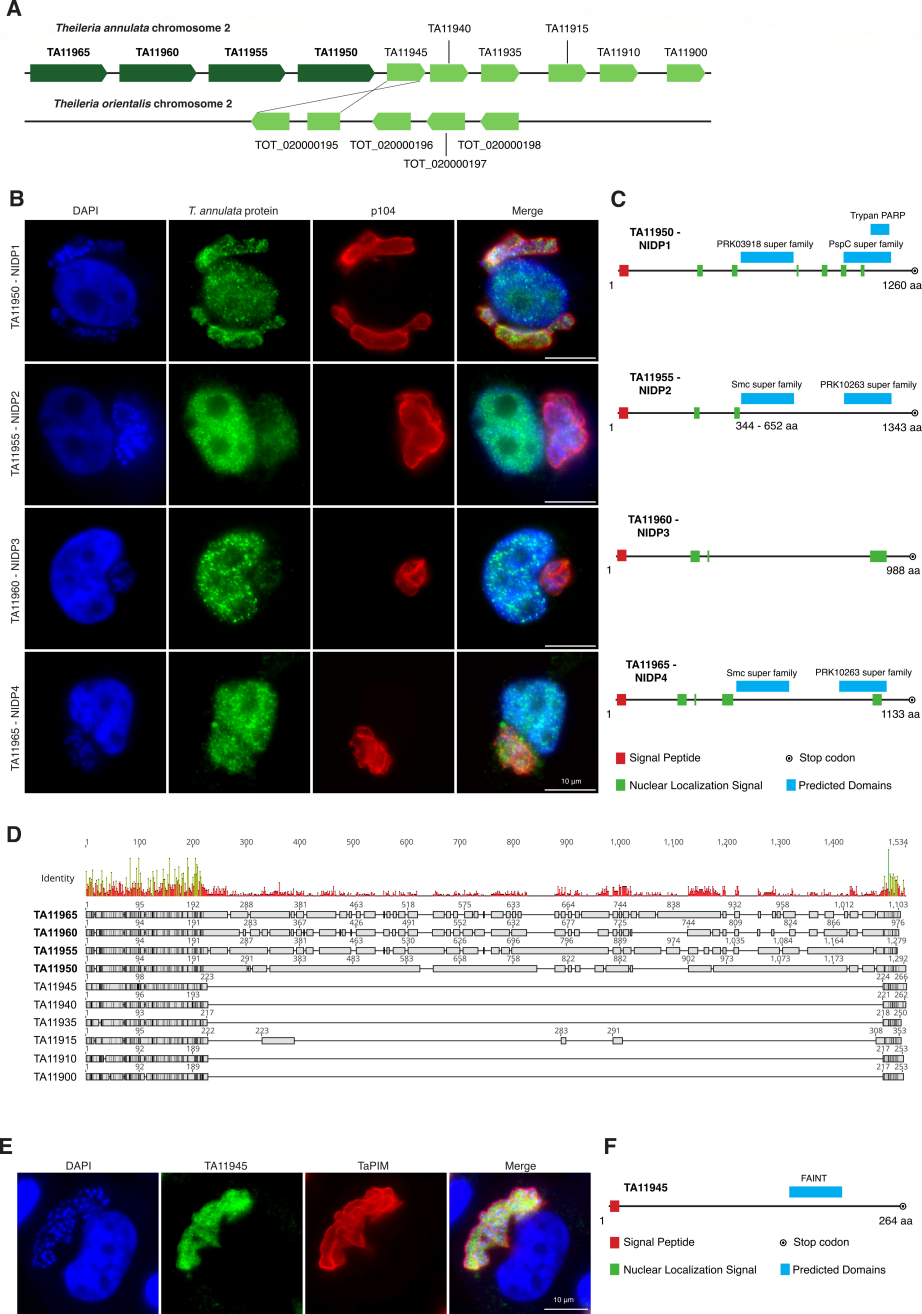
Characterization of a novel secreted NIDP family of *T. annulata* (**A**) Schematic of protein locus on chromosome 2 of *T. annulata* and *T. orientalis*, respectively. A phylogenetic analysis suggests that TA11945 is orthologs to TOT020000195. See also Fig. S2A. (**B**) TaC12 cells stained with α-TA11950 (NIDP1), TA11955 (NIDP2), TA11960 (NIDP3) and TA11965 (NIDP4), and α-p104 (schizont membrane) confirm the translocation of all four proteins into the host cell nucleus. Host cell nuclei and parasite nuclei are labeled with DAPI. (**C**) Schematic representation of proteins NIDP1–4 with predicted domains highlighted. (**D**) Alignment of *T. annulata* proteins NIDP1–4 with the other six members of the repetitive protein locus on a chromosome. (**E**) TaC12 cells stained with α-TA11945 and α-TaPIM (schizont membrane). The host cell nucleus and parasite nuclei are labeled with DAPI. (**F**) Schematic representation of protein TA11945 with predicted FAINT domain highlighted.

In the nucleus, we identified four additional, so far uncharacterized proteins (Fig. 1B). Subsequently, we conducted a bioinformatic search for commonalities among the identified exported proteins and discovered that all lacking an ortholog in *T. orientalis* exhibit a high intrinsically disordered protein structure, as predicted by IUPred3 (Fig. 1C) (49). Predictions by flDPnn (50) provided similar results (data not shown). The four so far undescribed *T. annulata* proteins in the nuclear fraction, TA11950, TA11955, TA11960, and TA11965, cluster in tandem repeats on chromosome 2 (Fig. 2A). Within this locus of 10 proteins, the four large proteins are flanked by an array of six considerably shorter genes (TA11945 to TA11900). Notably, phylogenetic analysis revealed that TA11945 bears the closest homology to TOT_020000195 in *T. orientalis*. The resulting phylogenetic tree showed a clustering of TA11950, TA11955, TA11960, and TA11965 on a separate branch distinct from the six smaller proteins in *T. annulata* and the four proteins present in *T. orientalis* (Fig. 2A; Fig. S2A).

We raised antibodies against TA11950, TA11955, TA11960, and TA11965, and confirmed their localization inside the host nucleus of the TaC12 cell line as well as inside the parasite schizont cytoplasm (Fig. 2B). Notably, no staining was observed in non-infected control cells or with pre-immune serum (Fig. S2B). Subsequently, we named the newly identified proteins *Theileria annulata* nuclear intrinsically disordered protein 1 (NIDP1; TA11950), NIDP2 (TA11955), NIDP3 (TA11960), and NIDP4 (TA11965). All four proteins contain a predicted signal peptide (SP) and predicted nuclear localization signals (NLS) (Fig. 2C). A domain search using the CDD/SPARCLE software (51) predicted the following conserved domains: For NIDP1, a homology to the PRK03918 superfamily (E-value: 9.77e−07) and a similarity to the trypan PARP region (E-value: 2.63e−10) and PspC superfamily (E-value: 5.00e−04) was found. The PRK03918 conserved protein domain family is found in the DNA double- strand break repair ATPase Rad50, a protein that is part of the structural maintenance of chromosome (SMC) protein family. Rad50 is also part of the MRN complex (MRN: complex consisting of MRE11, Rad50, and NBS1), which is implicated in DNA double-strand break repair (DBS), break recognition, and DNA end processing, and functions as a signal for cell cycle arrest (52). Both NIDP2 and NIDP4 contain a predicted domain for the conserved SMC superfamily (E-value NIDP2: 2.68e−09; NIDP4: 2.52e−04) as well as the PRK10263 superfamily (E-value NIDP2: 1.42e−03; NIDP4: 1.40e−05) (Fig. 2C). SMC proteins are necessary for chromosome condensation before mitosis, and in sister chromosome resolution and sister chromatid cohesion during mitosis. SMCs are also involved in DNA repair after mitosis and in the regulation of gene expression (53, 54). For NIDP3, no conserved domains were predicted. Taken together, the domain predictions suggest a potential involvement of three of the proteins in this family in the regulation of gene expression and chromosome maintenance.

To gain further insights into the overall protein structure of the arrayed protein family, we aligned the 10 members of the protein family and analyzed their amino acid similarity (Fig. 2D). Surprisingly, all members showed high sequence identities at the N-and C-terminal end (Fig. S2E). We used alphaFold2 predictions (55) of NIDP2 and TA11945 to further highlight the structural similarities between both proteins. Interestingly, whereas the N- and C-termini of NIDP2 overlap with TA11945, the center of NIDP2 (TA11955_252-1250_) has largely expanded (Fig. S2D and E), as has NIDP1, NIDP3, and NIDP4 (Fig. 2D). Large parts of these protein expansions appear highly disordered (Fig. 1C; Fig. S2F). Notably, TA11945 was detected within the parasite schizont only and appears not to get exported into the host cell (Fig. 2E). Unlike TaNIDP1–4, TA11945 harbors a FAINT domain (frequently associated with *Theileria* of unknown function) (48) and lacks an NLS (Fig. 2F).

### NIDP2 associates with host cell chromatin and is also found in the cytoplasm

**Fig 3:**
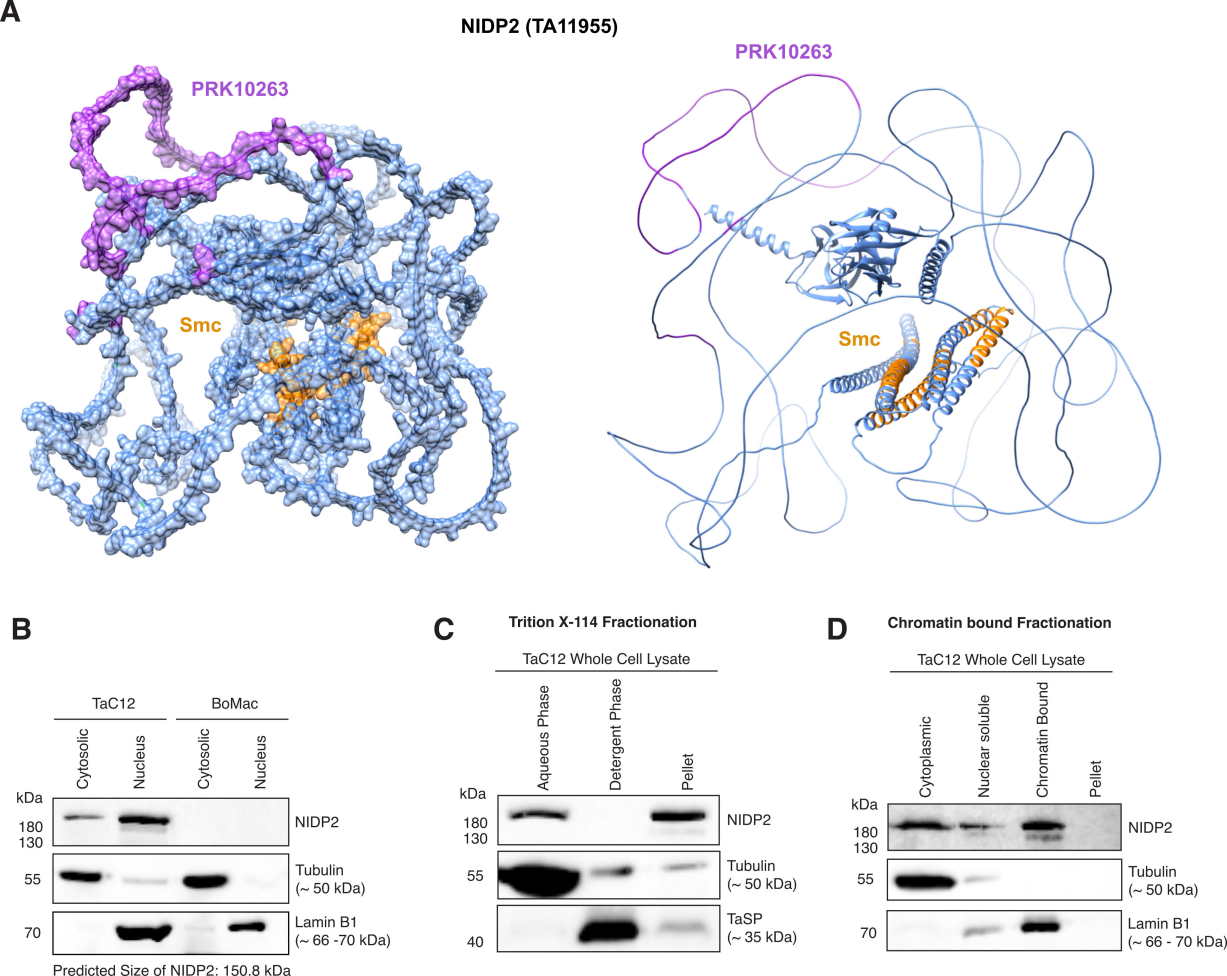
Characterization of NIDP2. (**A**) Predicted structure and domains of NIDP2 using alphaFold2. (**B**) Cytoplasmic and nuclear protein fractions of TaC12 and BoMac cells showed a stronger signal in the nuclear compared to the cytosolic fraction for NIDP2 >180 kDa. (**C**) Tac12 cells were fractioned with Triton X-114. NIDP2 could be detected in the aqueous phase and the pellet phase. Tubulin was used as a control for the aqueous phase while TaSP (TA17315), a Theileria surface parasite protein containing a transmembrane domain, functioned as a control for the detergent phase. (**D**) Cytoplasmic, nuclear soluble, chromatin-bound, and pellet fraction of TaC12 cells were analyzed by SDS-PAGE. NIDP2 was detected in the cytoplasmic, nuclear soluble, and chromatin-bound fractions. Tubulin was used as control for the cytoplasmic fraction and laminB1 for the chromatin-bound fraction.

NIDP2 is a highly disordered protein except for the N- and C-termini and a few alpha helices, which comprise the predicted SMC domain (Fig. 3A; Fig. S2E and F). Western blotting confirms that the protein is mainly localized in the host nucleus, with smaller amounts also detected in the cytoplasmic fraction (Fig. 3B). Although the predicted size of NIDP2 is 150.8 kDa, it is resolved with a higher apparent molecular weight of approximately 180 kDa by SDS-PAGE and Western blotting. Our IFA analyses indicate that proteins of the NIDP family might be expressed to some extent at the schizont surface (Fig. 2B). To investigate how NIDP2 interacts with the schizont membrane, we performed a Triton X-114 extraction and phase separation of TaC12 whole-cell lysates to separate hydrophilic from amphiphilic membrane proteins that become enriched in the detergent phase (56). We detected NIDP2 in both the aqueous phase and pellet fraction, in contrast to TaSP (TA17315) which, as a transmembrane protein, is enriched in the detergent fraction (57, 58) (Fig. 3C). This confirmed the prediction that NIDP2 contains no transmembrane domain, nor any GPI anchor. To determine whether NIDP2 in the pellet fraction is insoluble or chromatin associated, we fractionated the cells into cytoplasmic, nuclear, chromatin-bound, and pellet fractions (59). Immunoblotting revealed NIDP2 in the cytosolic, soluble nuclear, and chromatin-bound fraction, while the positive control Lamin B1 was predominantly found in the chromatin-bound fraction. This may indicate that NIDP2 can associate with the host chromatin (Fig. 3D). Of note, a slight double band is observable in the nuclear fraction and the chromatin-bound fraction (Fig. 3B and D) possibly indicating post-translational modification (PTM) of NIDP2 in the host nucleus, predominantly when associated with the chromatin. The sequence of NIDP2 contains several predicted phosphorylation sites (NetPhos 3.1), so to test the possible phosphorylation of NIDP2, we treated whole-cell lysates with lambda phosphatase prior to resolution by SDS-PAGE. A very slight shift in molecular weight indicated that NIDP2 is indeed likely to be phosphorylated, although compared to the *T. annulata* surface protein p104, previously shown to be phosphorylated (18), the degree of phosphorylation is rather small (Fig. S3B). Next, to test whether NIDP2, like p104, is phosphorylated in a cell-cycle-dependent manner, we synchronized cells in mitosis by nocodazole shake-off and resolved the lysate by SDS-PAGE in the presence of Phos-Tag, indicating a slight increase in phosphorylation during mitosis. Again, p104 was used as a control as we have previously shown this protein to be phosphorylated most significantly during mitosis (Fig. S3C) (18). In conclusion, NIDP2 is likely slightly phosphorylated, particularly during mitosis. We cannot exclude the contribution of other types of PTMs, such as deglycosylation.

### NIDP2 localizes to the schizont membrane via the CLASP1/CD2AP/EB1-complex in a cell-cycle dependent manner

**Fig 4:**
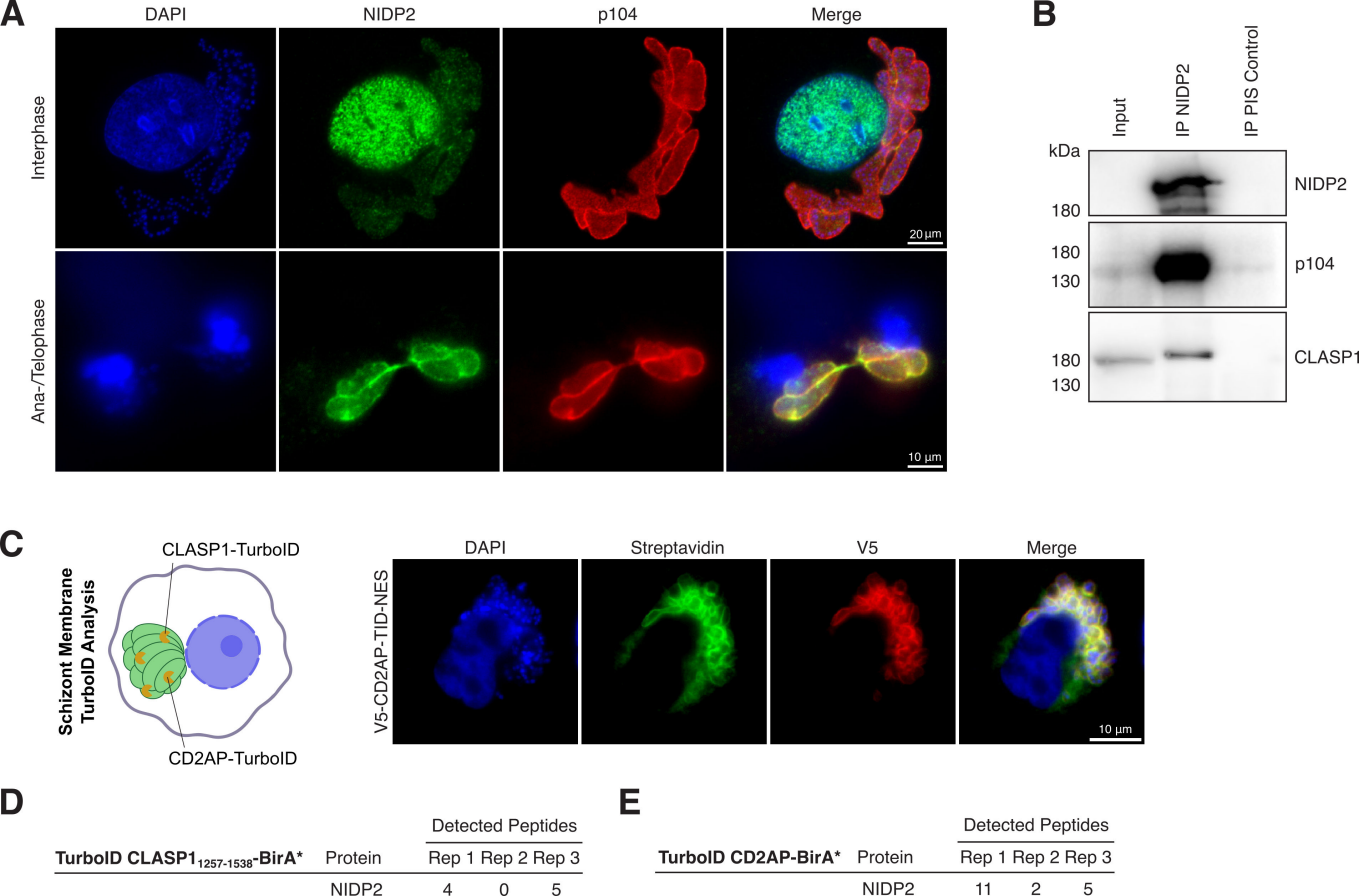
Cell-cycle-dependent localization of NIDP2 in host nucleus and CLASP1-CD2AP-EB1-p104-TaMISHIP parasite membrane complex (**A**) TaC12 cells stained with α-NIDP2 in interphase (upper panel) and ana-/telophase (lower panel). The schizont surface is stained with α-p104, and host and parasite nuclei with DAPI. Note the colocalization of p104 and NIDP2 during mitosis. See also Fig. S4. (**B**) Western blot analysis of NIDP2 immunoprecipitation (IP) from TaC12 cells (15% of total) blotted with primary antibodies as indicated. Preimmune serum (PIS) from the same rabbit served as control. Representative of three experiments with similar outcomes. (**C**) Schematic representation of TurboID analysis with TurboID-CD2AP and TurboID-CLASP1_1256−1538_ fusion proteins targeted to the parasite surface in TaC12 cells. Biotin-treated, V5-TID-NES-CD2AP-construct-transduced TaC12 cells show specific biotinylation of the parasite membrane (FITC-conjugated streptavidin; green). The host cell nucleus and parasite nuclei are labeled with DAPI. (**D**) CLASP1-associated NIDP2 peptides identified by LC-MS/MS analysis in three biological replicates. (**E**) CD2AP-associated NIDP2 peptides identified by LC-MS/MS analysis in three biological replicates. For clarity, only NIDP2 peptides are shown.

To further explore the potential function of the NIDP protein family, we decided to investigate their localization throughout the cell cycle of the host cell. Strikingly, NIDP2, but not NIDP1, NIDP3, and NIDP4 (not shown), localized exclusively to the parasite membrane during host cell mitosis, while all four family members are detected in the host nucleus during interphase (Fig. 4A; Fig. S3A). The biphasic localization of NIDP2 suggests a tightly regulated interaction with specific hosts and potentially other parasite proteins in two distinct compartments in a spatial and temporal manner. As the host cell enters mitosis and the host nuclear membrane breaks apart, NIDP2 colocalizes with the *T. annulata* protein p104 (18, 60) on the schizont surface (Fig. 4A; Fig. S3A). No residual staining of NIDP2 was observed close to or around condensed chromosomes until the host cell enters telophase/G1. The parasite membrane protein p104 has been shown to interact with host end-binding protein 1 (EB1) and CLIP-170-associating protein 1 (CLASP1) on the parasite surface. EB1 is an important regulator of MT dynamics and CLASP1 is a microtubule-stabilizing protein (16, 18). In addition to EB1 and CLASP1, the CD2-associated protein (CD2AP) can be found as part of this larger protein complex on the schizont surface (61). Notably, CLASP1 and CD2AP are present on the parasite surface during the whole-cell cycle of the host cell (16, 61). To further investigate the potential interaction of NIDP2 with the CLASP1/CD2AP/EB1-complex, we successfully coimmunoprecipitated p104 and CLASP1 together with NIDP2 in TaC12 cells (Fig. 4B). We found that we could co-precipitate NIDP2 with CLASP1 and p104 in both unsynchronized and mitotic cells (Fig. S3D). In addition, we engineered CD2AP-TurboID and CLASP1-TurboID constructs that target the schizont membrane throughout the cell cycle and stably expressed the fusion proteins in TaC12 cells (Fig. 4C; Fig. S3B). After subcellular protein fractionation, affinity-purified biotinylated proteins were analyzed by mass spectrometry in triplicates and the results were categorized as described before. NIDP2 was detected in both schizont-surface TurboID analyses (Fig. 4D and E). Our data therefore suggest that NIDP2 is a member of the EB1/CD2AP/CLASP1-complex. Unlike other parasite protein members of this complex such as p104 and MISHIP (61), NIDP2 translocates to the host nucleus during interphase. The interaction with the EB1/CD2AP/CLASP1 complex on the schizont surface appears to be transient and is not a result of NIDP2 integration into the parasite membrane (Fig. 3C).

### *In vivo* cross-linking of NIDP2 identifies proteins involved in cancer as potential host nuclear binding partners

**Fig 5:**
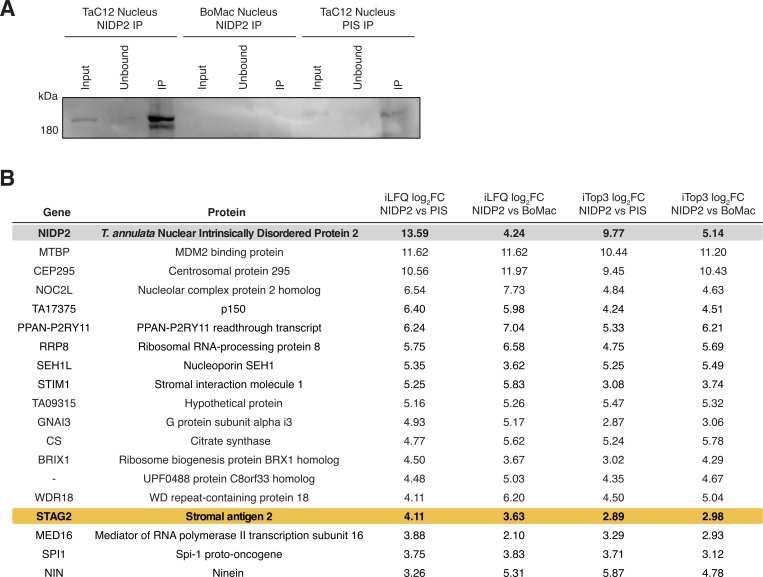
Identification of interaction partners of NIDP2 in the host nucleus. (**A**) Western blot analysis of nuclear fractions of TaC12 (infected) and BoMac (uninfected) cells immunoprecipitated (IP) with α-NIDP2; TaC12 cells were additionally probed with pre-immune serum (PIS) from the same rabbit. The Western blot was probed with α-NIDP2 as the primary antibody. (**B**) NIDP2-associated parasite and host proteins identified by LC-MS/MS analyses are shown. The iLFQ log_2_ fold change (FC) was calculated between three biological replicates of the NIDP2-IP of TaC12 cells and two biological replicates of the PIS-IP. The iLFQ log_2_ FC NIDP2 vs BoMac was calculated between three biological replicates of NIDP2-IP of TaC12 cells and two biological replicates of NIDP2-IP of BoMac cells. The iTOP3 log_2_ FC values were calculated in the same way.

To gain insights into the role of NIDP2 in the host nucleus of *T. annulata*-infected macrophages, we performed *in vivo* cross-linking and immunoprecipitated NIDP2 protein complexes in three biological replicates from nuclear extracts of TaC12 cells. As controls, we immunoprecipitated with rabbit pre-immune serum (PIS) from nuclear extracts of TaC12 cells and with α-NIDP2 from nuclear extracts of non-infected BoMac cell lysates (Fig. 5A). Protein complexes of all replicates and controls were analyzed by mass spectrometry (LC-MS/MS). Only proteins that were identified in α-NIDP2 pulldown assays from TaC12 cells, and not in the controls, were considered potential interactors of NIDP2 within the host nucleus. Aside from NIDP2, we did not pulldown any other members of the NIDP family.

As potential host-binding proteins, we identified multiple proteins implicated in cancers. Two proteins are implicated in the regulation of p53: Mouse double minute 2 (MDM2)-binding protein (MTBP) (62) and nucleolar complex protein 2 homolog (NOC2L) (63) (Fig. 5B). Importantly, we also identified stromal antigen 2 (STAG2), which serves as a tumor suppressor and an accessory protein of cohesin complexes (64). Cohesin, a protein complex associated with structural maintenance of chromosomes (SMCs), plays a critical role in sister chromatid cohesion, chromosome condensation, DNA repair, 3D genome organization, and gene expression, and is among the most commonly mutated protein complexes in cancer (65, 66).

### NIDP2 interacts with STAG2 in the nucleus of the host cell

**Fig 6:**
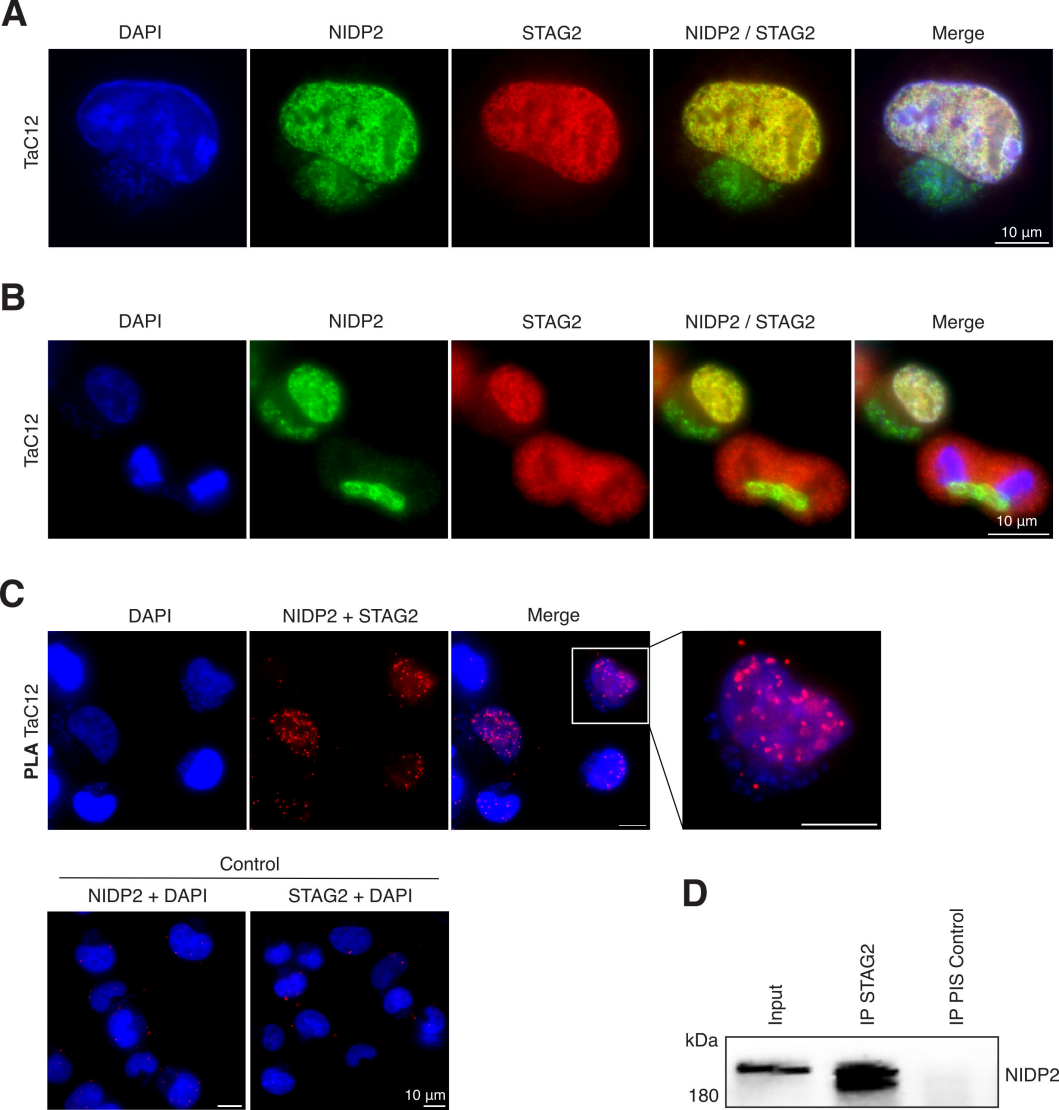
NIDP2 interacts with STAG2 in the host cell nucleus. (**A**) TAC12 cells stained with α-NIDP2 and α-STAG2 show colocalization of both proteins inside of the host nucleus, but not on the parasite surface. (**B**) The host cell in the upper left corner in (**B**) is in interphase, in the lower right corner in ana-/telophase. Host and parasite nuclei are stained with DAPI. (**C**) Proximity ligation assay (PLA) of TaC12 cells shows a signal in host cell nuclei only in the presence of both α-NIDP2 and α-STAG2. (**D**) Western blot analysis of STAG2 immunoprecipitation (IP) from TaC12 cells (20% of total) blotted with α-NIDP2. Preimmune serum (PIS) from the same rabbit served as control. Representative of three independent experiments with similar outcomes.

Given the predicted SMC domain for NIDP2 (Fig. 2C and 3A) and considering that STAG2 is frequently mutated in various cancers (67), we decided to investigate the NIDP2-STAG2 interaction further by IFA of both proteins in TaC12 cells. This revealed a high level of co-localization of both proteins within the host cell nucleus, while no such co-localization was found when NIDP2 was localized on the parasite surface during host cell division (Fig. 6A and B).

To further corroborate this finding, we utilized a proximity ligation assay (PLA) that produces a fluorescent signal when two proteins are within 40 nm of each other. This assay revealed a signal for the NIDP2-STAG2 antibody combination but no signal when both antibodies were applied alone (Fig. 6C), providing further evidence of the interaction of both proteins within the host cell nucleus. In line with this, we were also able to further confirm the interaction of STAG2 with NIDP2 by Western blot analysis after STAG2 immunoprecipitation from TaC12 cells (Fig. 6D).

## DISCUSSION

Transforming *Theileria* species uniquely induces a cancer-like state in infected host cells, marked by heightened proliferation, immortality, invasion, and metastasis. The specific parasite proteins and mechanisms driving these profound host cellular changes remain poorly understood, and, apart from bioinformatic predictions (34, 45, 48), an unbiased experiment to identify exported proteins has not been conducted. By employing a TurboID-based proximity labeling approach targeting different host compartments in *T. annulata-*infected macrophages, we identified several exported proteins and revealed a common feature among secreted *Theileria* proteins, namely a high protein disorder score, indicative of intrinsically disordered proteins (IDPs). In addition to known exported proteins, a novel protein family, nuclear intrinsically disordered proteins (NIDP) 1–4, clustered in tandem repeats on chromosome 2, was discovered. The identification of this new protein family, alongside members of the Ta9 and Tash families, underscores the significance of expanded protein families for transformative *T. annulata*. Our investigation into NIDP2 revealed an intriguing biphasic cell-cycle-dependent localization, demonstrating interactions with the EB1/CD2AP/CLASP1 complex on the parasite membrane during mitosis and with the tumor suppressor STAG2 within the host cell nucleus during interphase.

In our study, we focused on identifying parasite proteins exported to the host cell nucleus and cytoplasm. Alongside the newly identified NIDP1–4 protein family, the previously uncharacterized protein Tashb (TA03115) was found in the host nucleus. In addition, we confirmed the presence of known exported proteins: Ta9 (TA15705) in the host cytoplasm and TashAT2 (TA20095) in the host nucleus of TaC12 cells (36, 38). However, we did not detect other known exported proteins, such as TashHN (37) and TaPIN1. Most of the labeled proteins we detected in the host nucleus were, of course, of bovine origin. The presence of high-abundance bovine proteins likely limited the detection of low-molecular-weight or low-abundance *Theileria* proteins. It is noteworthy that a previous study in *Toxoplasma* using the alternative technique APEX2 (68), also encountered difficulties in identifying all known exported proteins in *Toxoplasma*, suggesting inherent limitations of the proximity labeling approach. Despite these challenges, our study underscores the efficacy of our TurboID-based approach in uncovering exported *Theileria* proteins, as demonstrated by the identification of Ta9, TashAT2, Tashb, and NIDP1-4. Notably, these proteins, which are absent from the genome of non-transforming *T. orientalis* (45), belong to larger gene families characterized by tandem repeats and variable copy numbers, further emphasizing the significance of our findings for understanding the *Theileria* exportome.

Genes critical for survival duplicate under selective pressure indicating adaptive evolution, resulting in copy number variation (69), possibly driven by their role in pathogenesis and invasiveness (70, 71). Examples from other protozoans, such as the *vsg, var,* and MEDLE gene families in trypanosomes*, Plasmodium* and *Cryptosporidium*, respectively, emphasize this evolutionary mechanism (72–74). Expanded gene families such as the *T. annulata* NIDP family are strikingly absent from the non-transforming *T. orientalis*. Notably, NIDP1–4 are tandemly arranged in a family of 10 proteins, with only one non-exported protein identifiable as an ortholog to a *T. orientalis* protein. The *T. orientalis* genome also harbors only a single-copy Tash gene. It is noteworthy that the ortholog of this gene in *T. annulata*, known as Tasha, is not expressed during the transformative schizont stage of the parasite (45). Furthermore, the *T. annulata* Ta9 gene shares only weak homology with the signal peptide region and C-terminal region of a *T. orientalis* gene (45). While both species infect leukocytes and develop into multinucleated schizonts, *T. annulata* induces uncontrolled lymphoproliferation prior to merogony, a feature absent in *T. orientalis*. Ultimately, our findings support the concept that extended gene families play a crucial role in the transformative capacity of *T. annulata* and may reflect the intimate host-pathogen co-evolution driven by an arms race between the parasite and its host (75).

In addition to the role of tandem arrayed proteins in malignant *Theileria*, the significance of intrinsically disordered proteins (IDPs) or intrinsically disordered regions (IDRs) in exported proteins among apicomplexans remains largely unexplored. For instance, *Toxoplasma* has numerous exported dense granule proteins such as GRA24, GRA16, and TgIST, all of which are characterized by distinct disordered protein structures (76–79). The structural flexibility and lack of a well-defined three-dimensional structure may allow for interaction with multiple host protein partners, potentially increasing functional complexity (12). The dynamic nature and rapid evolution of unstructured regions may also optimize the efficacy of the proteins in the parasite’s arsenal and potentially also influence trafficking across the parasitophorous membrane. Because IDRs lack a defined protein structure, they may facilitate a less energy-costly export of secreted proteins across the parasitophorous vacuole membrane (PVM), as they do not require unfolding to pass through a membrane channel such as the PTEX complex in *Plasmodium* (80). *Toxoplasma* effector proteins traverse the PVM *via* interaction with the putative MYR1 translocon (81), and the presence of structured tags impedes translocation, leading to protein entrapment within the parasitophorous vacuole (82). However, unlike *Toxoplasma* and closely related *Plasmodium*, *Theileria* (like *Babesia*) lacks a PVM, residing freely in a single membrane within the host cell’s cytosol, and no orthologs of the MYR1 or PTEX translocon protein members have been identified for *Theileria* (4). This suggests that protein export in *Theileria* occurs through an unrelated mechanism. Protein disorder may not be a prerequisite for membrane translocation into the host cell in *Theileria*. This notion is supported by the proteins TaPIN1 and TaPHB, two structured *T. annulata* proteins that have been identified to be exported into the host cell (34, 35). Hence, the IDP signature may not be essential for export but might reflect a rapid and evolutionarily cost-effective process for expressing novel interactors with versatile functions. This phenomenon is exemplified in the NIDP protein family. These differences in protein export mechanisms highlight the diversity in strategies employed by apicomplexan parasites for interacting with and manipulating their host cells. A comparative analysis of exported proteins in apicomplexans may provide valuable insights into the evolutionary advantage behind the IDP/IDR signature (83, 84).

Our structural analysis of NIDP proteins, especially NIDP1–4, indicates extensive disordered expansions between the N- and C-terminal conserved regions of the arrayed protein family. As less-defined protein structures are critical for the diverse functions of IDPs in key biological processes, such as signal transduction, transcriptional regulation, and cell cycle control (85), this feature may allow them to engage in promiscuous interactions with multiple protein binding partners. During interphase, NIDP2 localizes inside the host cell nucleus where stringent mass spectrometry analyses suggest multiple protein interaction partners including STAG2, mouse double minute 2 (MDM2)-binding protein (MTBP), and nucleolar complex protein 2 homolog (NOC2L), the latter two both involved in p53 regulation. We successfully validated the interaction of NIDP2 and STAG2, a cohesion complex member and well-established cancer gene associated with various malignancies, including acute myeloid leukemia and bladder cancer (67). Notably, both p53 and MDM2 regulation have been previously shown to be altered in *Theileria*-infected cells (31, 32). Unfortunately, attempts to confirm the interaction of NIDP2 with MTBP and NOC2L were inconclusive due to the unreliable performance of commercially available antibodies in the bovine background.

Notably, during mitosis, NIDP2 relocates to the schizont membrane and interacts with the EB1/CD2AP/CLASP1 complex, which is involved in microtubule interaction and further interaction with parasite proteins p104 and TaMISHIP (4, 61). This raises intriguing questions about NIDP2’s dual function on the parasite surface and in the host cell nucleus, as well as its potential role in microtubule binding during mitosis. While attempts to ectopically express truncated forms of NIDP2 in bovine cells were unsuccessful, further work is needed to determine the function of NIDP2 in the nucleus, which regions of the protein interact with the EB1/CD2AP/CLASP1 complex, and whether NIDP2 interacts with additional proteins, as suggested by our mass spectrometry data set.

In conclusion, by employing an unbiased TurboID-based proximity labeling approach, we identified a set of exported proteins characterized by a predicted high protein disorder score, notably the newly identified NIDP family, alongside the established Ta9 and Tash protein families. These findings challenge simplistic assumptions regarding the sole significance of protein disorder in facilitating protein export over the PVM in apicomplexan parasites. Instead, they suggest the existence of additional functional implications for the evolutionary development of protein disorder in apicomplexans. The detailed analysis of NIDP2’s biphasic cell-cycle-dependent localization and interactions, including its association with the tumor suppressor and cohesion protein STAG2 and the EB1/CD2AP/CLASP1 membrane complex, sheds new light on previously unknown versatile dynamics of exported *Theileria* proteins. Collectively, these discoveries establish a foundation for further investigations into the molecular mechanisms governing *Theileria*-induced cancer-like host cell alterations.

## MATERIALS AND METHODS

### Maintenance of mammalian cell lines

TaC12 (*T. annulata* infected macrophage cell line), BoMacs (non-infected bovine macrophage cell line) (86), and HEK293T were cultured in flasks and well plates from TPP (Techno Plastic Products AG, Switzerland). BoMacs and HEK293T were kept in DMEM at 37°C and 5% CO_2_ atmosphere. TaC12 was cultured in L15 at 37°C in the absence of CO_2_ as described previously (18). The culture media from Gibco were supplemented with 10% fetal calf serum (FCS; BioConcept, Allschwil, Switzerland; Cat. No. 2-01F10-I), 2 mM L-glutamine (BioConcept, Cat. No. 5-10K00-H), 100 IU/m penicillin, and 100 µg/mL streptomycin (BioConcept, Cat. No. 4-01F00-H), 10 mM HEPES pH 7.2 (Merck, Darmstadt, Germany; CAS-No: 7365-45-9). TaC12 cells were washed with PBS and incubated with 1 mM PBS/EDTA (Merck) and the remaining cell lines were washed with PBS and incubated with 1× trypsin-EDTA PBS (BioConcept, Cat. No. 5-51K00-H) until detachment. Cells were split with culture media as required.

### Expression constructs

Primers used in this study were purchased from Microsynth AG (Balgach, Switzerland), and constructs were verified by Sanger sequencing (Microsynth AG). Cloning was performed using either Gibson Assembly (New England Biolabs, NEB, Ipswich, MA) or the In-Fusion HD cloning kit (Takara Bio, San Jose, CA), following the manufacturer’s instructions. Plasmids were transformed into NEB 5-alpha competent *E. coli*, while lentiviral constructs were transformed into Endura cells (BioCat, Heidelberg, Germany). Plasmids obtained from Addgene (Watertown, MA, USA) are listed in the supplement (Table S2). Lentiviral constructs, including pNLS-TurboID (p1141), pNES-TurboID (p1140), CD2AP-TurboID (p1138), and pCLASP11256-1538-TurboID (p1139) utilized pLenti CMV GFP Puro (#17488) as a linearized template. For GST fusion proteins, TA11950_2203-2545_, TA11955_1642-1974_, TA11960_1249-1617_, and TA11965_1375-1698_ were amplified from TaC12 genomic DNA and cloned into the pGST-parallel3 vector (see Table S2 for details).

### Lentiviral transduction and FACS sorting

HEK239T cells were utilized to produce lentiviruses and were transfected with FuGENE HD transfection reagent (Promega, Madison, WI; Cat. No. E2311) using a third-generation lentiviral transfer vector system as described (16). Briefly, the gene of interest containing plasmid pRRL-RSrII, along with packaging vector psPAX2 and envelope vector pMD2.G (Table S2), were transfected into HEK293T cells in a 5:3:2 ratio. Twenty-four hours post-transfection media were replaced, and lentiviral-particle-containing media were harvested 48 h and 72 h post-transfection. Prior to transduction, the harvested media were filtered through a 45-µm filter membrane. TaC12 wild-type (WT) cells (2 × 10^5^) were transduced with 5 mL of the collected virus-containing media. The transduction of TaC12 cells was performed twice within a 48-h period, with a recovery time of 24 h between both transduction steps. TaC12 cells expressing NES, NLS, CLASP1_1256−1538_- and CD2AP-TurboID constructs were sorted into a 96-well plate as single cells using the FACS sorter Aria III (BD Biosciences, San Jose, CA, USA).

### Antibodies

GST fusion proteins (GST-TA11950_2203-2545_, GST-TA11955_1642-1974_, GST-TA11960_1249-1617_, and GST-TA11965_1375-1698_) were overexpressed in BL21 Star *E. coli* (Invitrogen ThermoFisher Scientific, Waltham, MA). The proteins were purified using glutathione Sepharose beads (GE Healthcare, Waukesha, WI) and subsequently sent to Eurogentec (Seraing, Belgium) for antibody production in rabbits. Peptides from Tashb (aa sequence: CQYVKSDSDNEENNND) and TA11945 (aa sequence: CEGVTESGELYSKSTY) were synthesized by Eurogentec and used for immunization in rats (Eurogentec, Seraing, Belgium).

### Immunofluorescence assays

Cells were seeded onto glass coverslips and incubated overnight and either treated or remained non-treated prior to fixation with 4% PFA for 15 min at room temperature (RT) before washing with PBS and permeabilization in 0.2% Triton X‐100 (diluted in PBS) for 10 min. Subsequently, cells were blocked in 10% FCS in PBS for 1 h at RT. Alternatively, cells were fixed with ice-cold methanol, washed twice with PBS, and blocked in 10% FCS in PBS for 1 h at RT. Primary antibodies were diluted in 10% heat‐inactivated FCS in PBS and put directly onto the cells for 1 h at RT. After primary antibody staining, cells were washed five times in PBS and secondary antibodies were diluted in 10% heat‐inactivated FCS in PBS and incubated for 1 h at RT. DNA was stained using DAPI (Invitrogen), and samples were mounted onto slides by using mounting media (DAKO), if not mentioned otherwise. Freshly prepared samples were either analyzed on a DeltaVision Elite system (GE Healthcare) equipped with Olympus IX‐70 inverted microscope and a CMOS camera, using a 100× Olympus Objective, and software from SoftWorx (Applied Precision) or an Eclipse 80i microscope (Nikon) equipped with a Hamamatsu Orca R2 camera using a 100× PlanApo objective (Nikon) and the OpenLab 5 software (Improvision). The PLA assay was performed according to the manufacturer’s instructions using the *In Situ* Detection Reagents Red (Catalogue Number: DUO92008, Merck, Darmstadt, Germany).

### TurboID to identify proteins interacting with CLASP1, CD2AP and located in the host cytoplasm and nucleus

TaC12 cells stably transduced with the described TurboID-fusion constructs NLS-TurboID, NES-TurboID, CD2AP-TurboID, and CLASP1_1256−1538_-TurboID were incubated in media containing 500 µM Biotin (Serva) for 3 h (CLASP1_1256−1538_ is the minimal region for CLASP1 that still allows it to bind to the parasite membrane (16). As control the same cell lines were incubated for 3 h without Biotin. Cells were washed with PBS prior to detachment. Proteins were extracted using the NE-PER Kit (Thermo Scientific, Catalog Number 78833) for NLS-TurboID (nuclear fraction) and NES-TurboID (cytosolic fraction) and the Subcellular Protein Fractionation Kit (Thermo Scientific, Catalog Number 78840) for CD2AP-TurboID and CLASP1_1256−1538_-TurboID (membrane fractions). After protein extraction, lysates were incubated overnight at 4°C with Pierce Streptavidin Magnetic Beads (Thermo Scientific, Catalog Number 88816) which were washed beforehand with RIPA lysis buffer (50 mM Tris [pH 8], 150 mM NaCl, 0.1% SDS, 0.5% sodium deoxycholate, 1% Triton X-100, complete EDTA-free protease inhibitor [Roche, Basel, Switzerland]). Subsequently, the magnetic beads were washed once with 1× 1 M KCl, 1× 0.1 M Na_2_CO_3_, 1× 2 M urea in 10 mM Tris-HCl (pH 8) and twice in RIPA lysis buffer. The supernatant was removed and beads were snap-frozen prior to mass spectrometry and immunoblotting.

### *In vivo* cross-linking and protein complex isolation

Ten million TaC12 cells were harvested and washed with PBS. Subsequently, the cells were incubated with 0.1% (wt/vol) paraformaldehyde (PFA) in PBS for 8 min at RT to cross-link the proteins. To stop the reaction, glycine was added at a final concentration of 125 mM and incubated for 5 min at RT. After this, the cells were washed in PBS. The cell pellets were put on ice and resuspended in ice-cold lysis buffer (20 mM Tris [pH 7.5], 140 mM KCl, 1.8 mM MgCl_2_, 0.1% NP-40, 10% glycerol) containing complete protease inhibitor cocktail EDTA free (Roche) and sonicated 3 times for 10 s at 10% power with a Branson Digital Sonifier with 30 s intervals. After centrifugation at 16,000 × *g* for 5 min, the lysate was put on lysis buffer-washed Pierce Protein A Magnetic Beads (Catalog number: 88845) and Pierce Protein G Magnetic Beads (Catalog number: 88847) together with rabbit and rat or mice antibodies, respectively, incubated together prior for 6 h at 4°C. The lysates were further incubated overnight at 4°C and subsequently washed thrice with RNP lysis buffer. Finally, cross-linking reversal and elution of proteins were performed by incubation with 1× Lämmli buffer for 20 min at 95°C and analyzed by mass spectrometry and immunoblotting.

### Mass spectrometry analysis of streptavidin and antibody-based protein pull-downs

For mass spectrometry of streptavidin pull-downs, the beads were incubated in 50 µL 3× reducing LDS sample buffer containing 15 mM DTT and 2 mM biotin at 95°C for 10 min prior to loading the entire sample onto a BoltTM 12% Bis-Tris-Plus gel and briefly running them into the top of the gel. The gel was fixed and stained with colloidal Coomassie blue G250 stain (17% [wt/vol] ammonium sulfate, 34% methanol, 0.5% acetic acid, 0.1% [wt/vol] Coomassie blue G-250), subsequently reduced and alkylated, and then washed to remove SDS and stain before digestion with trypsin (500 ng) overnight at 37°C. Peptides were extracted from the gel pieces, dried down, and samples were re-dissolved in 2.5% acetonitrile and 0.1% formic acid. 5 µL of each digest was run by nanoLC-MS/MS using a 2-h gradient on a 0.075 mm × 250 mm C18 column feeding into a Q-Exactive HF mass spectrometer. All MS/MS samples were analyzed using Mascot (Matrix Science, London, UK; version 2.6.2). Mascot was set up to search the Bos_taurus_Refseq_002263795.1_ARS-UCD1.2_20190510.fasta (63687 sequences) and cRAP_20150130.fasta (123 sequences; contaminant file) for the three searches, plus one more database for each as described: (i) Old database (OldDB)—Theileria_annulataAnkara_PiroplasmaDB-43_AnnotatedProteins_20190510 database (3,796 entries), (ii) Uniprot—uniprot-Theileria_annulata_refproteome_UP000001950_20190508 database (3790 entries), (iii) New database (NewDB)—Theileria_annulataAnkara_PiroplasmaDB-43_AnnotatedProteins_20191214 database (3,572 entries). The searches were done assuming the digestion enzyme trypsin. Mascot was searched with a fragment ion mass tolerance of 0.060 Da and a parent ion tolerance of 10.0 PPM. Deamidated of asparagine and glutamine, oxidation of methionine, and carbamidomethyl of cysteine were specified in Mascot as variable modifications. Scaffold (version Scaffold_4.8.9, Proteome Software Inc., Portland, OR) was used to validate MS/MS-based peptide and protein identifications. Peptide identifications were accepted if they could be established at greater than 80.0% probability by the Peptide Prophet algorithm (87) with Scaffold delta-mass correction. Protein identifications were accepted if they could be established at greater than 99.0% probability and contained at least one identified peptide. Protein probabilities were assigned by the Protein Prophet algorithm (88). Proteins that contained similar peptides and could not be differentiated based on MS/MS analysis alone were grouped to satisfy the principles of parsimony. Proteins sharing significant peptide evidence were grouped into clusters.

For mass spectrometry of proteins that were immunoprecipitated by antibodies as bait, washed protein fractions were loaded on SDS-PAGE gel, fixed in methanol-acetic acid-water (45:1:54) for 20 min, and subsequently stained with colloidal Coomassie staining and digested as described above. The digests were analyzed by liquid chromatography LC-MS/MS (PROXEON coupled to a QExactive mass spectrometer, ThermoFisher Scientific, Reinach, Switzerland) with one injection of 5 µL digests. Peptides were trapped on a µPrecolumn C18 PepMap100 (5 µm, 100 Å, 300 µm × 5 mm, ThermoFisher Scientific, Reinach, Switzerland) and separated by backflush on a C18 column (5 µm, 100 Å, 75 µm × 15 cm, C18) by applying a 40 min gradient of 5% acetonitrile to 40% in water, 0.1% formic acid, at a flow rate of 350 nL/min. The Full Scan method was set with a resolution of 70,000 with an automatic gain control (AGC) target of 1E06 and a maximum ion injection time of 50 ms. The data-dependent method for precursor ion fragmentation was applied with the following settings: resolution 17,500, AGC of 1E05, a maximum ion time of 110 ms, mass window 2 *m*/*z*, collision energy 27, under fill ratio 1%, charge exclusion of unassigned and 1+ ions, and peptide match preferred, respectively. The mass spectrometry data were then searched with MaxQuant (89) version 1.6.14.0 against the following concatenated databases: *Theileria annulata* strain Ankara (PiroplasmaDB, release 43), *Theileria annulata* TaC12 deNovo proteins (manuscript in preparation; no Masking), and uniprot (UniProt Consortium, 2019) *Bos taurus* (release 2021_03), to which common potential contaminants were added. The digestion enzyme was set to trypsin with a maximum of three missed cleavages, peptide tolerance for the first search to 20 ppm, and the MS/MS match tolerance to 25 ppm. Carbamidomethylation on cysteine was given as a fixed modification, while methionine oxidation, asparagine, and glutamine deamidation as well as protein N-terminal acetylation were set as variable modifications. Match between runs were allowed between replicates. Identification filtering was controlled by a false discovery rate set at 0.01 at both peptide-spectrum match and protein level. Proteins with one-peptide identification were allowed. Next to MaxQuant’s Label-Free Quantification (LFQ) values, protein abundance was also obtained by adding the intensities of the top three most intense peptides (Top3) (90), after normalizing the peptide forms by variance stabilization (91). Imputation was performed at the peptide form level for Top3 (iTop3), and the protein level for LFQ (iLFQ). In either case, missing values were replaced by a draw from a Gaussian distribution if there was at most one non-missing value in a group of replicates; this distribution was such that its width was 0.3× sample standard deviation and centered at the sample distribution mean minus 2.8 or 2.5× sample standard deviation, for, respectively, peptide or protein level. Any remaining missing values were imputed by the Maximum Likelihood Estimation (92) method.

### Cell synchronization, fractionation and isolation of chromatin-bound proteins

Cellular fractionation was performed as described above or with Triton X-114 as described previously (56). Briefly, 2 mL of Triton X-114 (Fluka, BioChemika, 93422) was resuspended in 98 mL PBS and dissolved at 0°C and incubated overnight at 30°C. The next day the upper aqueous phase was removed and PBS was added to the same volume as removed and again dissolved at 0°C. This procedure was repeated two more times to obtain 10% condensed Triton X-114. For cell fractionation, a 1 mL 1% Triton X-114 solution was made and mixed with pellets of 10 × 10^6^ TaC12 cells and resuspended by vortexing and sonication 3 × 10 s at 10% power. After centrifugation at 16,000 × *g* for 5 min, the supernatant was removed, and the pellet was put aside. The supernatant was warmed at 37°C for 1 min (until the solution became cloudy) and then spun at 775 RCF for 1 min and the upper aqueous phase was removed from the lower detergent phase. Both phases were precipitated by the methanol-chloroform procedure (93) and all fractions were resuspended in 1× Lämmli buffer. The isolation of chromatin-bound proteins was performed as described previously (59). To synchronize cells in mitosis, TaC12 cells were arrested in prometaphase by incubating with 0.1 µg/mL nocodazole (Biotrend) for 16 h prior to harvest by shake-off. For removal of phosphate groups prior to SDS-PAGE analysis, lysates were prepared in the absence of the phosphatase inhibitor and treated with lambda protein phosphatase (NEB) following the manufacturer’s instructions. To separate phosphorylated proteins, lysates were analyzed by SDS-PAGE in the presence of 20 µM Phos-tag acrylamide (NARD institute) following the manufacturer’s instructions.

### Protein structure predictions

To predict the structure of the identified proteins, IUPred3, flDPnn, and AlphaFold2 were used (49, 50, 55, 94). For IUPred3, the analysis type was set to long disorder and medium smoothing. For AlphaFold2, the Multiple Sequence Alignment (MSA) mode utilized was MMseqs2, incorporating UniRef and environmental sample sequence databases (UniRef + Environmental). The number of models was set to 5, with 24 recycles. Convergence of recycles occurred in all models before reaching 24. The relax max iterations were set to 200, and the pairing strategy was set to greedy.

## SUPPLEMENTAL MATERIAL

Fig. S1 mbio.03412-23-s0001.tiff TurboID controls; validation of TashAT2, Tashb, and Ta9 protein localization in TaC12 cells; and alignment of Tash and Ta9 locus, related to Figure 1.

Fig. S2 mbio.03412-23-s0002.tiff Additional controls and analyses of NIDP1-4 and TA11945 proteins, related to Fig. 2.

Fig. S3 mbio.03412-23-s0003.tiff Localization of NIDP2 during interphase and mitosis; TurboID controls.

Legends mbio.03412-23-s0004.docx Legends for supplemental figures and tables.

Table S1 mbio.03412-23-s0005.xlsx Identified parasite proteins in host cell by TurboID proximity labeling.

Table S2 mbio.03412-23-s0006.xlsx Vector constructs used in this study.

ASM does not own the copyrights to Supplemental Material that may be linked to, or accessed through, an article. The authors have granted ASM a non-exclusive, world-wide license to publish the Supplemental Material files. Please contact the corresponding author directly for reuse.
