# Supplementary figures and images for: TurboID mapping reveals the exportome of secreted intrinsically disordered proteins in the transforming parasite Theileria annulata
Source: mBio. 2024 May 15;15(6):e03412-23. doi: 10.1128/mbio.03412-23 (PMC11237503; doi:10.1128/mbio.03412-23)

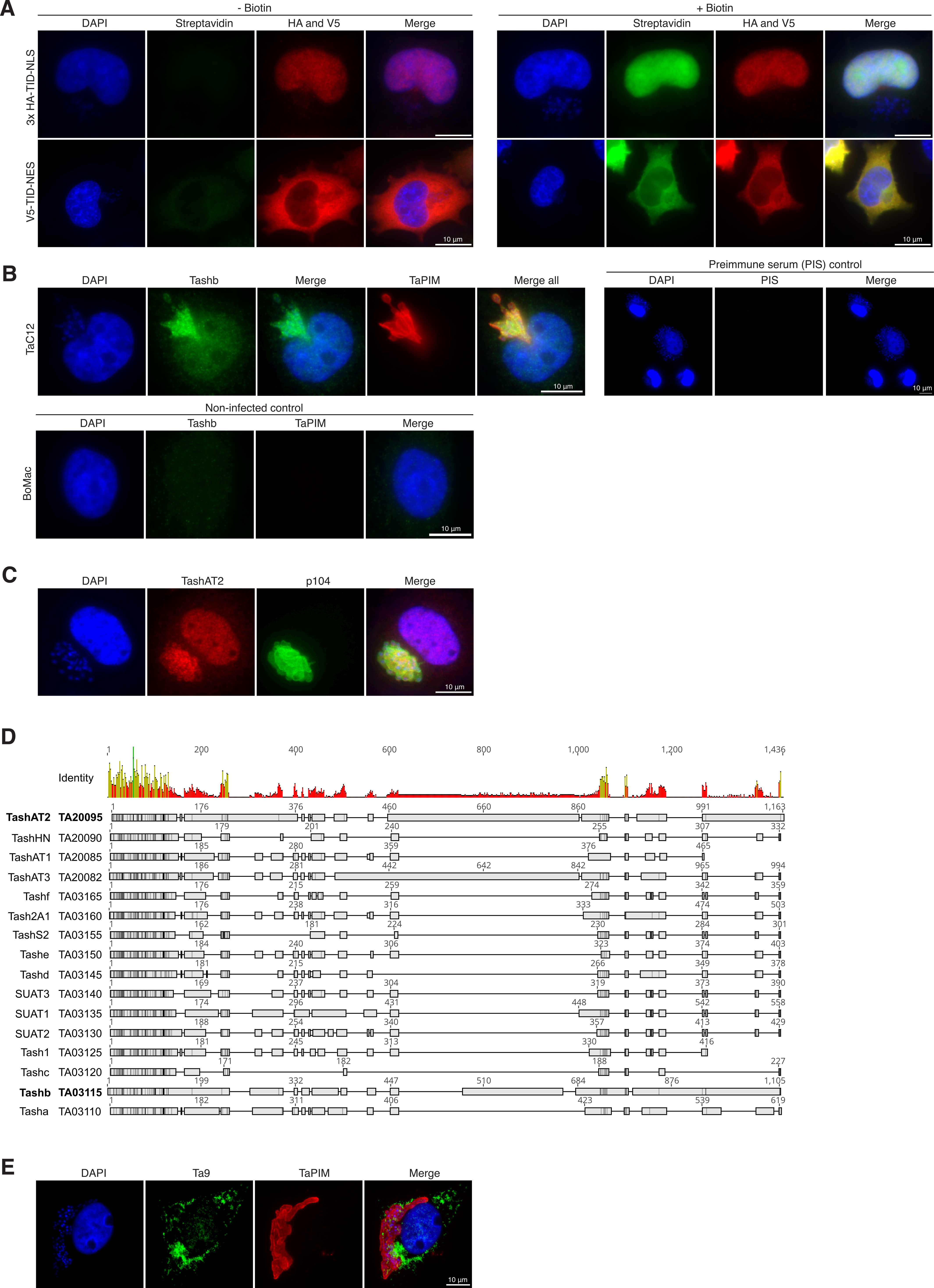

Supplement: Fig. S1 — TurboID controls; validation of TashAT2, Tashb, and Ta9 protein localization in TaC12 cells; and alignment of Tash and Ta9 locus, related to Figure 1. [file mbio.03412-23-s0001.tiff]

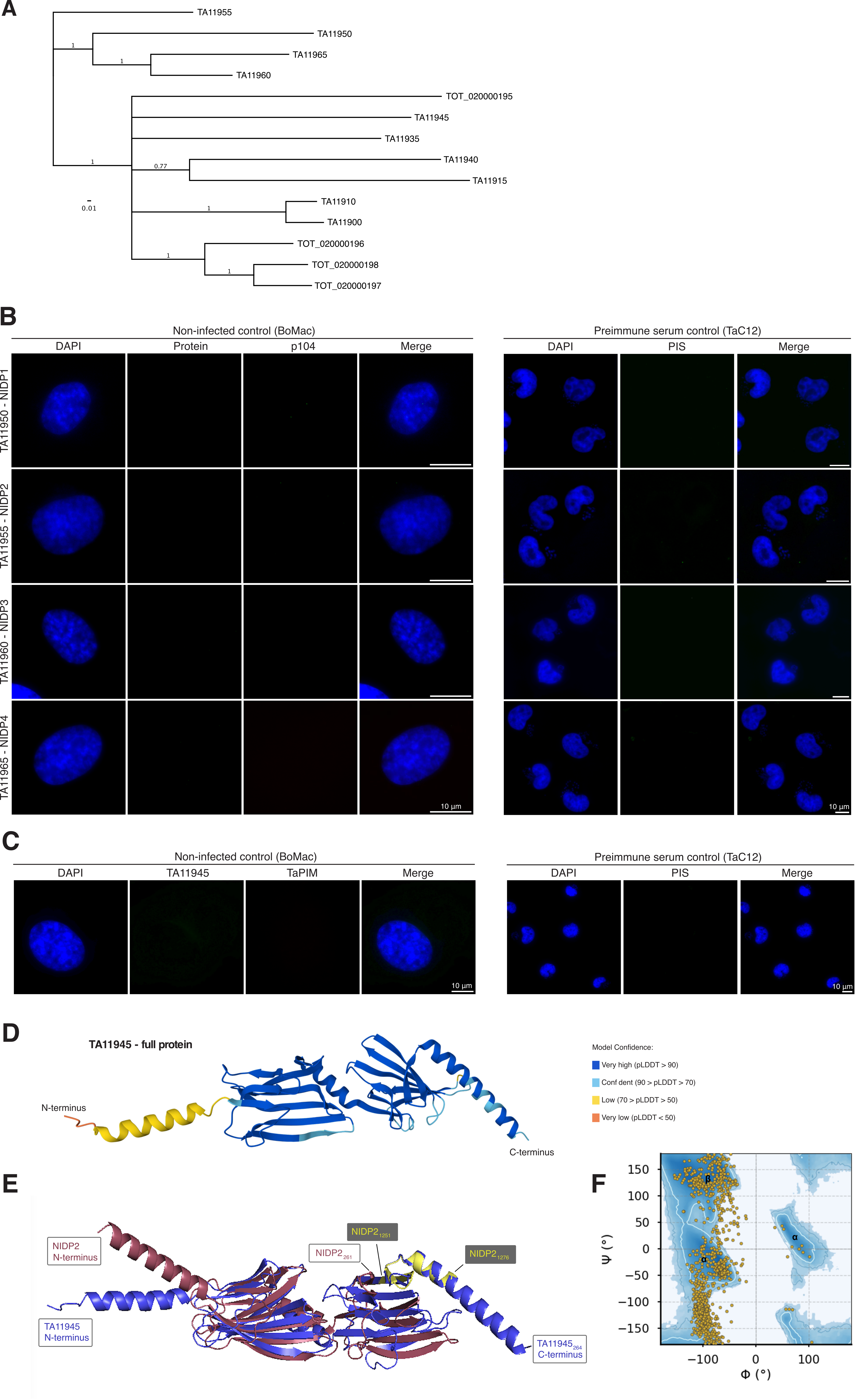

Supplement: Fig. S2 — Additional controls and analyses of NIDP1-4 and TA11945 proteins, related to Fig. 2. [file mbio.03412-23-s0002.tiff]

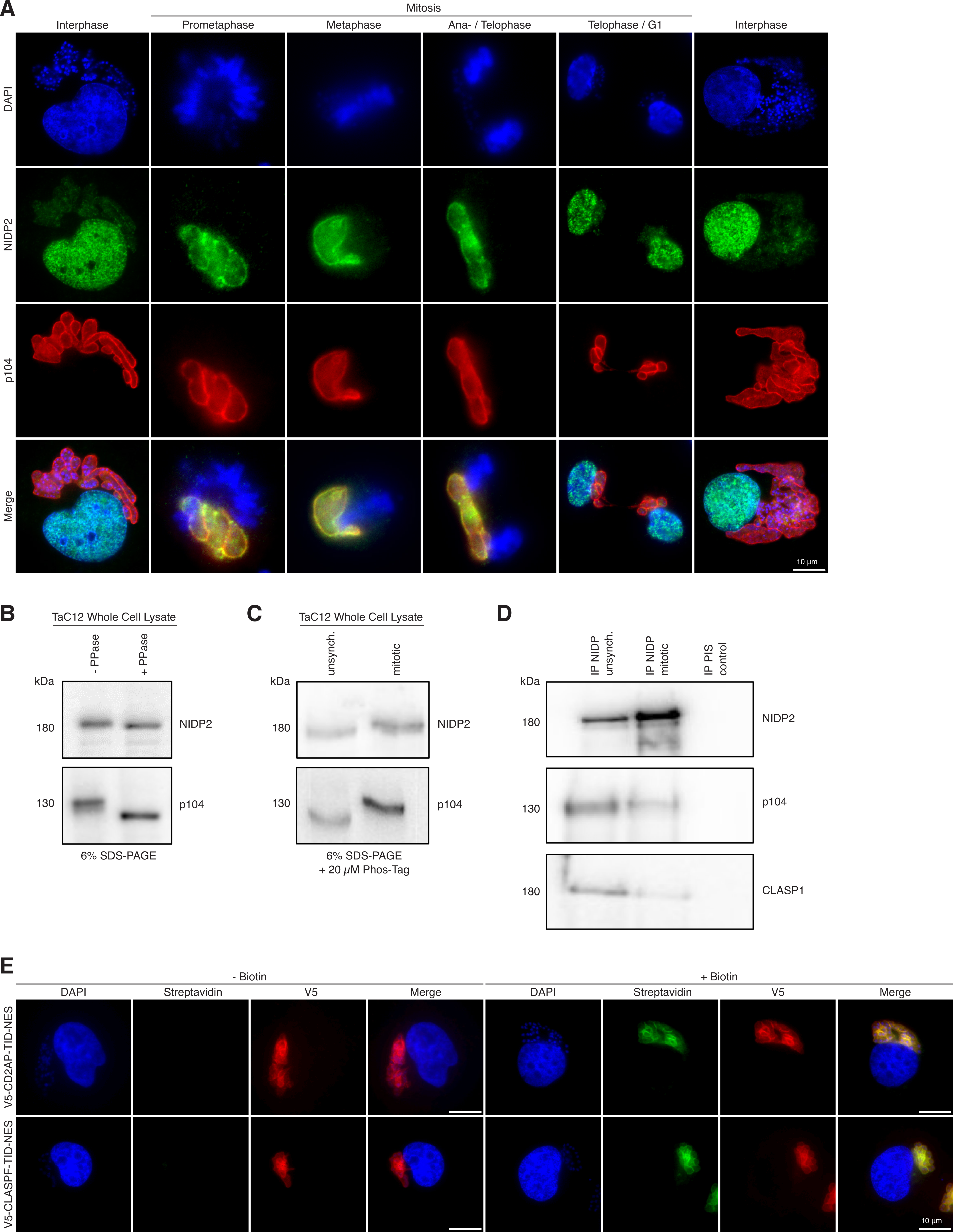

Supplement: Fig. S3 — Localization of NIDP2 during interphase and mitosis; TurboID controls. [file mbio.03412-23-s0003.tiff]
