# Supplementary material for: TurboID mapping reveals the exportome of secreted intrinsically disordered proteins in the transforming parasite Theileria annulata
Source: mBio. 2024 May 15;15(6):e03412-23. doi: 10.1128/mbio.03412-23 (PMC11237503; doi:10.1128/mbio.03412-23)
Supplement: Legends — for supplemental figures and tables. [file mbio.03412-23-s0004.docx]

**SUPPLEMENTAL FIGURE AND TABLE LEGENDS**

**Figure S1. TurboID controls, validation of TashAT2, Tashb and Ta9 protein localization in TaC12 cells, and alignment of Tash and Ta9 locus, related to Figure 1**

**(A)** TaC12 cells transduced with HA-TID-NLS and V5-TID-NES constructs, respectively, incubated without (left) and with (right) 150 µM biotin for 30 min prior to fixation. Analysis with α-HA and α-V5 confirms the correct cellular localization of the biotin ligase (red) and enzyme activity (FITC-conjugated streptavidin). Host cell and parasite nuclei are labelled with DAPI. (**B)** TaC12 cells stained with rat α-Tashb (TA03115) and α-TaPIM (schizont membrane). Host cell and parasite nuclei are labelled with DAPI. Right panel shows control stained with pre-immune serum (PIS) of the same rat. Host cell and parasite nuclei are labelled with DAPI. Lower panel shows α-Tashb and α-TaPIM staining of noninfected control cells (BoMac). **(C)** TaC12 cells stained with α-TashAT2 and α-p104 (schizont membrane). Host cell and parasite nuclei are labelled with DAPI. **(D)** Alignment of T. annulata TashAT2 and Tashb with the other 14 proteins of the Tash locus. **(E)** TaC12 cells stained with α-Ta9 and α-TaPIM (schizont membrane). Host cell and parasite nuclei are labelled with DAPI.

**Figure S2. Additional controls and analyses of NIDP1 – 4 and TA11945 proteins, related to Figure 2**

**(A)** Phylogenetic analysis of identified protein family members of T. annulata and T. orientalis. Note that TA11950, TA11955, TA11960 and TA11965 cluster distinct from T. orientalis and other T. annulata proteins of this gene cluster, and TA11945 in closest proximity to T. orientalis TOT_20000195. **(B)** As controls, non-infected BoMac cells were stained with α-NIDP1 - 4 and α-p104, and TaC12 cells were stained with corresponding rabbit pre-immune sera (PIS). Nuclei were labelled with DAPI. **(C)** As controls for TA11945, non-infected BoMac cells were stained with α-TA11945 and α-p104, and TaC12 cells were stained with the corresponding rat pre-immune serum (PIS). Nuclei were labelled with DAPI. **(D)** Predicted structure of TA11945 by alphaFold2. **(E)** Overlay of N-terminus and C-terminus of NIDP2 and entire protein structure of TA11945. (**F**) The Ramachandran plot of NIDP2 shows that most of the residues take up sterically permitted regions that represent disordered regions.

**Figure S3. Localization of NIDP2 during interphase and mitosis, TurboID controls**

**(A)** TaC12 cells stained with α-NIDP2 in interphase and mitosis. The schizont surface is stained with α-p104, and host and parasite nuclei with DAPI. During prometaphase, metaphase and ana-/telophase NIDP2 colocalizes with p104. In interphase and during telophase/G1 phase NIDP2 localizes in the nucleus of the host cell. (**B**) Western blot analysis of TaC12 lysates treated with lambda phosphatase, blotted with anti-NIDP2 or anti-p104 (as a control) antibodies. (**C**) Western blot analysis of lysates from unsynchronized or mitotic TaC12 cells in the presence of 20 μM Phos-Tag, blotted with anti-NIDP2 or anti-p104 (as a control) antibodies. (**D**) Western blot analysis of NIDP2 immunoprecipitation (IP) from unsynchronized or mitotic TaC12 cells (15% of total) blotted with primary antibodies as indicated. Preimmune serum (PIS) from the same rabbit served as control. **(E)** Transduced cell lines with V5-CD2AP-TurboID-NES and V5-CLASPF-TurboID-NES (CLASPF = CLASP1_1256−1538_) were fixed with PFA and analyzed by immunofluorescence analysis (IFA). V5-tagged-TID was labeled with anti-V5 to confirm the correct localization of the biotin ligase. V5-CD2AP-TurboID-NES and V5-CLASP1_1256−1538_-TurboID-NES cells were incubated with 150 µM biotin for 30 min prior to fixation and analyzed with FITC-conjugated streptavidin. Host cell nucleus and parasite nuclei are labelled with DAPI.

**Table S1. Identified parasite proteins in host cell by TurboID proximity labeling**

**Table S2. Vector constructs used in this study**
